# Supplementary material for: Live-cell GLUT4 translocation assay reveals Per3 as a novel regulator of circadian insulin sensitivity in skeletal muscle cells
Source: Biol Open. 2025 Jul 18;14(7):bio061941. doi: 10.1242/bio.061941 (PMC12309902; doi:10.1242/bio.061941)
Supplement: Supplementary information [file biolopen-14-061941-s1.pdf]

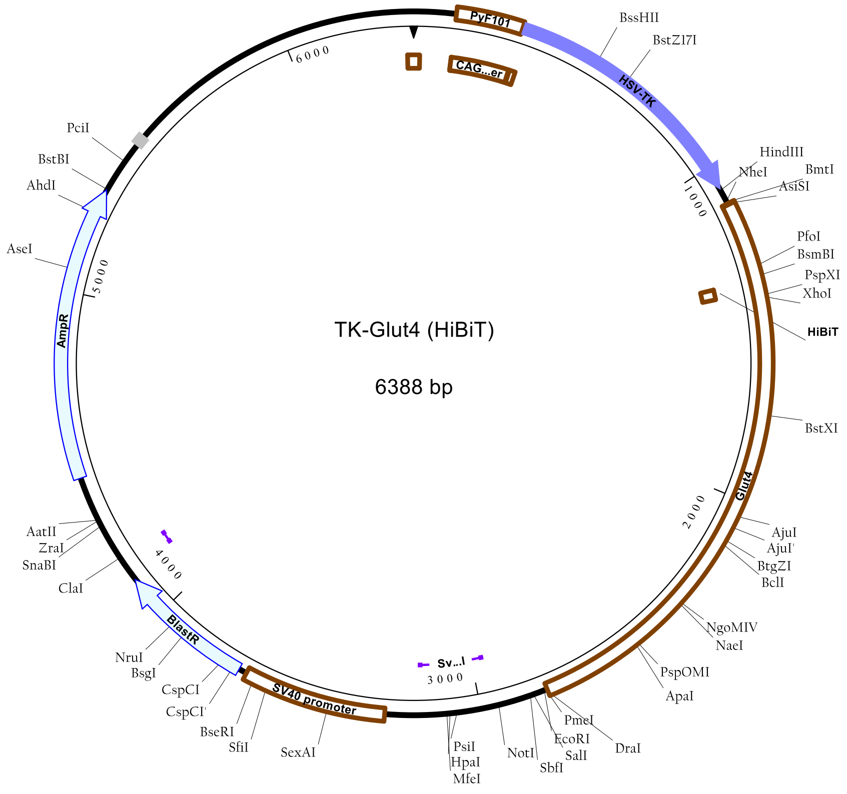

Fig. S1. Map of the TK-Glut4 (HiBiT) mammalian expression plasmid (6,388 bp–Promega UK limited).

(i) A synthetic CAG promoter (brown box) drives the herpes-simplex-virus thymidine-kinase gene (**HSV-TK**; blue arrow), followed by the pYF101 poly-adenylation signal. HSV-TK functions as a conditional “suicide” gene for negative selection.
(ii) A coding sequence for the human facilitative glucose transporter 4 fused at its C terminus to the 11-amino-acid HiBiT luminescent tag (**Glut4–HiBiT**; orange arrow).

For selection, the plasmid encodes **AmpR** (β-lactamase; blue arrow) for propagation in *E. coli* and **BlastR** (blasticidin-S deaminase; blue arrow) driven by the SV40 promoter (brown box) for stable selection in mammalian cells. Purple arrowheads mark SV40 and synthetic poly-adenylation sites. Unique restriction-enzyme sites are shown around the perimeter; enzymes used for cloning or linearisation in this study are underlined in the text. Arrow direction indicates the orientation of transcription. The map was generated with SnapGene v.7.1 and is drawn to scale.

Biology Open • Supplementary information

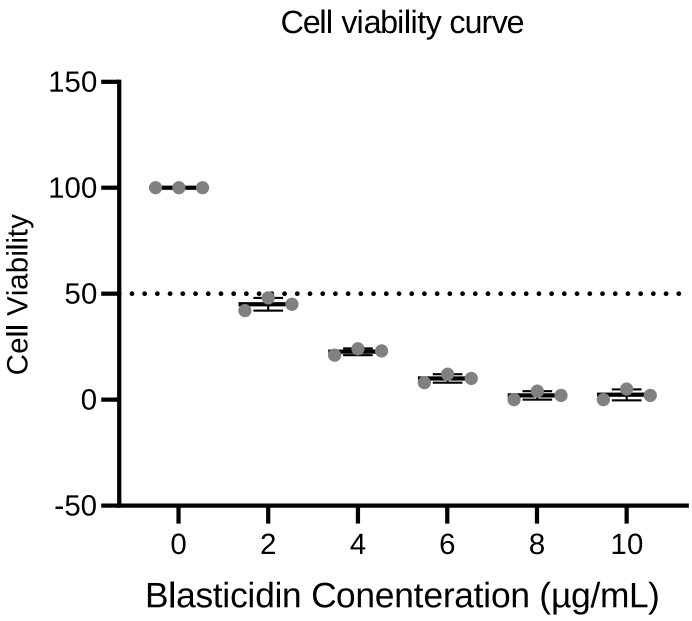

Fig. S2. Cytotoxicity of blasticidin in cultured cells.

Cultured cells were incubated with the indicated concentrations of blasticidin (0–10 µg ml^{-1}). After treatment, cells were harvested, mixed 1:1 with 0.4 % (w/v) Trypan-blue solution and viable (dye-excluding) versus non-viable cells were enumerated in a haemocytometer. Grey circles show individual biological replicates (n = 3 per dose) expressed as % viable cells relative to the untreated control; black horizontal bars denote the mean. The dotted line indicates 50 % viability, corresponding to an IC_{50} of ~2.5 µg ml^{-1} under these conditions. Results are representative of three independent experiments and are plotted as mean ± s.d.

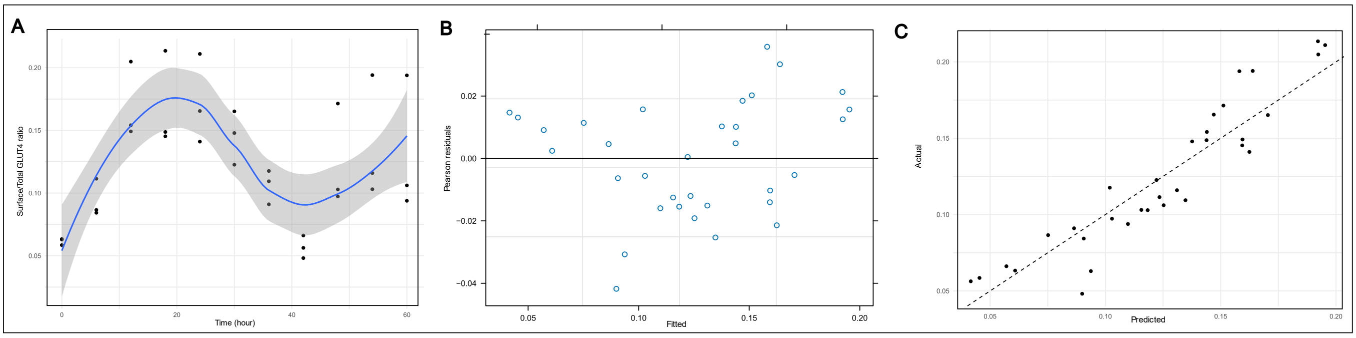

Fig. S3. GLUT4 translocation efficiency over time. (A) Time-dependent GLUT4 translocation measured as the surface-to-total GLUT4 ratio following insulin stimulation (30 nM). Points indicate individual observations at each time point, and the solid blue line represents the LOESS-smoothed trend with the shaded area indicating a 95% confidence interval. (B) Model diagnostics showing Pearson residuals plotted against fitted values from the mixed-effects model. Random distribution around zero indicates the validity of the model assumptions. (C) Predicted vs. actual GLUT4 translocation ratios from the mixed-effects model. The dashed diagonal line represents perfect prediction (y = x), demonstrating strong predictive accuracy of the model.
